# StAP1 phage: an effective tool for treating methicillin-resistant *Staphylococcus aureus* infections

**DOI:** 10.3389/fmicb.2023.1267786

**Published:** 2023-09-29

**Authors:** Yuwen Lu, Yifei Lu, Baobao Li, Jiazhen Liu, Lixin Wang, Lianyang Zhang, Yang Li, Qiu Zhong

**Affiliations:** ^1^Department of Laboratory Medicine, Daping Hospital, Army Medical University, Chongqing, China; ^2^State Key Lab of Trauma, Burn and Combined Injury, Medical Center of Trauma and War Injury, Daping Hospital, Army Medical University, Chongqing, China; ^3^State Key Lab of Trauma, Burn and Combined Injury, Chongqing Key Laboratory for Disease Proteomics, Institute of Burn Research, Southwest Hospital, Army Medical University, Chongqing, China; ^4^General Hospital of Eastern Theater Command, PLA, Nanjing, China

**Keywords:** methicillin-resistant *Staphylococcus aureus*, bacteriophage, a broad host range, phage therapy, *Herelleviridae* phage

## Abstract

**Introduction:**

*Staphylococcus aureus* infection has long been a serious concern in the medical field, with methicillin-resistant *Staphylococcus aureus* (MRSA) posing a considerable challenge to public health. Given the escalating bacterial resistance and the favorable biosafety and environmental properties of phages, the resurgence of phage therapy offers a promising alternative to antibiotics.

**Methods:**

In this study, we isolated and characterized a MRSA phage named StAP1 from a Chinese hospital. Phenotypic and molecular analyses revealed its broad-spectrum characteristics, genomic background, and potential application in MRSA infection treatment.

**Results:**

Morphological examination classified the phage as a member of the *Herelleviridae* phage family, displaying a typical hexagonal head and a slender fibrous tail. Genomic analysis unveiled a size of ~144,705 bp for the StAP1 genome, encompassing 215 open reading frames (ORFs). The one-step growth curve demonstrated a 20-min incubation period for the phage, with an optimal multiplicity of infection (MOI) of 0.1. Moreover, StAP1 exhibited stability across a wide range of temperatures and pH levels. Further investigation of its broad-spectrum characteristics confirmed its ability to effectively infect all staphylococcal cassette chromosomal mec (SCCmec) types found in MRSA strains, notably displaying a remarkable lysis rate of 76.7% against the prevalent ST239 strain in China. *In vivo* studies show cased significant efficacy of the StAP1 phage against MRSA infection.

**Discussion:**

Overall, StAP1 phage presents a broad infection spectrum and exhibits strong lytic effects on various MRSA strains, highlighting its tremendous potential as a powerful tool for MRSA infection treatment.

## Introduction

*Staphylococcus aureus* (*S. aureus*), a representative pathogenic staphylococcus, often causes a wide range of human infections, from minor skin infections to severe tissue infections and life-threatening sepsis (Lowy, 1998; Tong et al., 2015). With the extensive use of antibiotics, the generating selective pressures enable *S. aureus* to evolve with various resistance mechanisms against almost all first-line antimicrobials (De Oliveira et al., 2020; Magalhães et al., 2021). Dominated by horizontal transfer of the SCC*mec* and their high diversity, *S. aureus* acquired resistance against methicillin and most β-lactam antibiotics, thus developing into methicillin-resistant *S. aureus* (MRSA) (Peacock and Paterson, 2015; Hassoun et al., 2017; Partridge et al., 2018). Since the late 1980s, MRSA has been the most clinically prominent pathogen due to its tachytelic evolution and global dissemination. The prevalence of MRSA has presented a critical challenge for global healthcare settings and clinical therapy (Lakhundi and Zhang, 2018; Turner et al., 2019). According to the molecular typing surveys of MRSA, several major clones, such as ST1, ST5, and ST239, have mediated expanding dissemination across continents and serious infections with high mortality (Chambers and Deleo, 2009; Bal et al., 2016; Lakhundi and Zhang, 2018). Therefore, the development of an alternative therapeutic approach against MRSA infections is urgently desired.

Bacteriophages (phages), which are natural viral predators of bacteria, possess the ability to lyse target hosts (Salmond and Fineran, 2015). As a promising strategy at the forefront of treating recalcitrant MRSA infections, phage therapy has been revitalized in the post-antibiotic era (Gordillo Altamirano and Barr, 2019; Petrovic Fabijan et al., 2020). Despite the great potential of phage therapy using virulent phages to combat MRSA infections, there is little evidence provided in preclinical and clinical studies that phages have robust efficacy as full-fledged antibacterial agents (Uyttebroek et al., 2022). The high specificity of phages provides unique advantages, while their narrow host range accompanying high personalization becomes a major obstacle in utilizing phage-based applications. The use of a cocktail of phages contributes to overcoming this limitation to a certain extent; hence, appropriate selection and combination are essential for phage therapy (Kortright et al., 2019; Strathdee et al., 2023). Even though various MRSA phages have been well-characterized, efficient phages with wide host ranges that target clinical MRSA strains are still an urgent need. Phages with a broad host range are not only ideal candidates for phage therapy but also offer and enrich the options for the phage resource (Moller et al., 2019; Strathdee et al., 2023).

In this study, we isolated and identified a phage named StAP1 from the sewage of Southwest Hospital with a clinical MRSA strain recovered from a patient with burns as the host. The phenotypic and molecular profiles of StAP1 were then characterized, which was a broad host range against clinical MRSA (which infects 46.3% of 162 tested clinical MRSA strains). Moreover, StAP1 exhibited excellent therapeutic effects against the tissue infection of MRSA. This study shows that StAP1 has the potential to be a novel avenue for combating the growing severity of MRSA infections and broadens the pavement of phage therapy.

## Methods and materials

### Bacterial strain and culture condition

The clinical MRSA strain XN61 was recovered from burn patients who were hospitalized at the Institute of Burn Research, Southwest Hospital of Army Medical University (Chongqing, China) in 2019 and used as a host for phage enrichment. Species and antimicrobial susceptibility profiles were identified via both 16S rRNA gene sequencing and the VITEK-2 compact system (bioMérieux, France) following the manufacturer's instructions. The obtained primary strain was kept in our laboratory in a−80°C medium containing 20% (w/v) glycerol. This strain was cultivated at 37°C in brain heart infusion (BHI) broth medium (Oxoid, United Kingdom) with constant shaking for 6 h to reach the log phase.

### The isolation and purification of phage

According to the previously reported method (Jiang et al., 2020), StAP1 was isolated from sewage samples collected from Southwest Hospital, utilizing MRSA strain XN61 as the primary phage propagation host. In brief, the filtered sewage sample was incubated overnight with 200 ml of BHI medium and 100 ml of XN61 in the log phase. The mixture was centrifuged at 10,000 × *g* for 10 min to collect the supernatant, which was then gradiently diluted to test the formation of phage plaques using double-layer plates.

Once the plaques were formed, phage purification was conducted by repeated picking and plating of single plaques based on plate lysate and the polyethylene glycol (PEG) 8000-NaCl precipitation method (Govind et al., 2006). The details were as follows: DNase I and RNase A were added to the mixture at a final concentration of 1 μg/ml at 37°C for 30 min to degrade the nucleic acid completely. NaCl was added at a concentration of 5.84 g/100 ml and then incubated on ice for 1 h. PEG #8000 was dissolved in the collected supernatant at a final concentration of 10% w/v and further kept on ice at 4°C overnight. Finally, the resulting precipitate was dissolved in 2.5 ml of TM solution (0.05 mol/L Tris-Cl, pH 7.5, 0.2% MgSO_4_.7H_2_O). An equal volume of chloroform was used to remove PEG #8000 and obtain a high-concentration phage supernatant. The phages were stored at 4°C for subsequent experiments.

### Morphological characterization

The morphology of StAP1 was revealed and observed by transmission electron microscopy (TEM). The purified phage particles were first dialyzed in normal saline. Then, a total of 20 μl samples were fixed with 2% glutaraldehyde for 30 min and spotted onto a carbon-coated copper grid. After absorbing for 5 min, excessive samples were removed using a filter paper strip. The phage particles were then negatively stained with 2% (w/v) potassium phosphotungstate (pH 7.0) for 2–3 min and observed with a transmission electron microscope (Shimadzu, Japan).

### DNA extraction, sequencing, and bioinformatics

DNA of StAP1 phage particles was extracted via the standard phenol-chloroform extraction method (Lu et al., 2013). DNase I (5 μg/ml) and RNase A (1 μg/ml) were added to the purified phage and incubated at 37°C for 1 h to degrade DNA and RNA from the host bacteria. Proteinase K (50 μg/ml) and SDS (0.5%) were added to the mixture, mixed well, and incubated at 56°C for 1 h to lyse the phage capsid and release the nucleic acid. Afterward, an equal volume of balanced phenol (pH = 8.0) was added, and the mixture was centrifuged at 5,000 × *g* for 10 min. The upper aqueous phase was collected and mixed with an equal volume of chloroform, followed by centrifugation at 5,000 × *g* for 10 min. The upper aqueous phase was further mixed with 0.6 times the volume of isopropanol. After centrifuging, the obtained precipitate was washed once with 70% ethanol and absolute ethanol, respectively. To obtain phage genomic nucleic acid, an appropriate amount of TE (0.5 ml) was utilized to dissolve the precipitate.

For the complete sequences of the genome, DNA subjected to whole genome sequencing (WGS) was sequenced by Shanghai Personalbio Technology Co., Ltd. using the Illumina NovaSeq High-throughput Sequencing Platform (Illumina, United States), with sequencing depth = 100-fold with a 100-bp paired-end. Illumina sequence reads were then *de novo* assembled in contigs through SPAdes (github.com/ablab/spades). RAST (rast.nmpdr.org) and Bakta (github.com/oschwengers/bakta) were employed to add the annotation and predict open reading frames (ORFs) (Aziz et al., 2008; Schwengers et al., 2021). Gene structures were visualized and analyzed using SnapGene V 4.1.8. The complete nucleotide sequence of StAP1 was deposited in GenBank under accession number OQ025229.

For comparative genomics, the BLAST Ring Image Generator (BRIG) (brig.sourceforge.net/) was used to conduct multiple comparisons of genome sequences available at the National Center for Biotechnology Information (NCBI), and a composite circular map was generated (Alikhan et al., 2011). The evolutionary tree of phage StAP1 and its close reference phages was constructed and visualized using the Virus Classification and Tree Building Online Resource (VICTOR) (ggdc.dsmz.de/victor.php), which was based on the Genome-BLAST Distance Phylogeny (GBDP) method (Meier-Kolthoff and Göker, 2017).

### One-step growth assay

According to the reported method, 10 ml of XN61 was grown in BHI broth to a density of OD_600_ = 0.5 in the early log stage. Purified phage particles were incubated with the host bacteria to achieve a MOI of 0.1 in a total volume of 2 ml, and 10 min was allowed for adsorption. The mixture was then centrifuged at 10,000 × *g* for 1 min and washed with BHI broth three times to remove the free phages. Resuspended cells with adsorbed phage were transferred to 2 ml of BHI at 37°C with shaking at 120 rpm. A total of 100 μl of supernatant were recovered periodically at 0, 10, 20, 30, 40, 50, 60, and 70 min and employed to perform phage plaque assays against XN61. This assay was repeated three times with duplicate samples to obtain the one-step growth curve, based on which the burst size and time were calculated.

### Optimal multiplicity of infection (MOI)

To determine the optimal MOI of phage StAP1, purified phage stock solution was continuously diluted and incubated with an equal number of MRSA cells (10^8^ PFU/ml) until the host bacteria were completely lysed. After centrifuging at 10,000 × *g* for 10 min, phage titers of the supernatant were determined through the double-layer agar plate method. The optimal MOI was calculated as the ratio that produced the highest phage titer. The experiment was performed in triplicate to ensure accuracy.

### PH and thermal stability of stap1

To investigate the effect of pH on phage stability, 100 μl of StAP1 (10^10^ PFU/ml) was mixed with 900 μl of PBS at gradient pH levels (ranging from pH 3.0 to 12.0) and incubated at 37°C for 120 min. After 10-fold dilution, 10 μl of each diluted sample was, respectively, incubated with 200 μl of host bacteria for 10 min and added to 5 ml of 0.75% BHI agar. The phage titers were calculated using the double-layer agar plate method. Similarly, for the thermal stability assay, aliquots were exposed to different temperatures (4, 25, 37, 4, 50, 60, and 70°C) for 60 min.

### Host range determination

For host range determination, a total of 228 non-duplicate clinical isolates (162 MRSA and 66 methicillin-sensitive *S. aureus* isolates) were collected in 2009 in our previous study (Cheng et al., 2013). For MRSA strains, the minimal inhibitory concentration (MIC) of oxacillin was tested at more than 4 μg/ml according to the Clinical and Laboratory Standards Institute (CLSI, 2018) document M100-S28. STs were identified by the MLST database (https://pubmlst.org/organisms/staphylococcus-aureus), and the SCC*mec* types were verified *via* SCC*mec*Finder 1.2 (https://cge.food.dtu.dk/services/SCCmecFinder/). Briefly, an equal volume of cultivated bacteria (OD_600_ around 0.5) and phage was mixed and spotted onto BHI agar plates, with 5 μl of bacteria without phage serving as a control. The plates were then incubated overnight and investigated for bacterial growth to assess whether phage StAP1 was able to lyse the bacteria. Plates with no bacterial growth were seen as being extremely lysed, whereas plates with a small number of colonies and many colonies were regarded as being moderately lysed and non-lysed, respectively.

### *In vivo* MRSA infection experiments

Male BALB/c mice (6–8 weeks; 20–25 g) were purchased from the Animal Center of the Army Medical University. All experimental procedures involving animals were approved by the Laboratory Animal Welfare and Ethics Committee of Army Medical University (No. AMUWEC20237406) and carried out in strict accordance with ethical principles. To investigate the *in vivo* antibacterial effect of StAP1, the subcutaneous abscess model was established, referring to our previous report (Lu et al., 2021). In brief, mice were anesthetized with 1% sodium pentobarbital solution (50 mg kg^−1^, intraperitoneally), and then 100 μl suspension of MRSA (1 × 10^8^ CFU/ml) was subcutaneously injected into the two sites on the shaved back of each experimental mouse. A focal MRSA infection was induced as a subcutaneous abscess after 24 h.

The successfully modeled mice were randomly divided into two groups. The experimental group was administrated 100 μl of phage StAP1 (1 × 10^9^ CFU/ml), while the control group received a subcutaneous injection of an equal volume of PBS. StAP1 and PBS were administered once every 2 days, and observations were carried out every 48 h for a total of 6 days.

A round green card with a 6-mm diameter was displayed as the reference for the initial abscess area. A digital camera was utilized to record the abscess area on the day of infection. The abscess area was quantified and analyzed using the ImageJ (NIH) software, and a curve of the abscess area of the mouse back skin infection was plotted. Additionally, abscess tissues were collected on day 6, fixed with 4% paraformaldehyde, and embedded in paraffin for sectioning into 5-μm thick sections. Hematoxylin and eosin (H&E) staining was performed for histological analysis. The abscessed tissues were also extracted and ground for colony counting.

### Statistical analysis

Data are presented as means ± SD of at least three or more independent measurements. Statistical analysis was performed through a two-tailed Student's *t*-test or one-way ANOVA using SPSS 18.0 (IBM, United States). Tukey's *post-hoc* test was utilized for multiple *post-hoc* comparisons after a one-way ANOVA. *P* < 0.05 was considered significant. Graph analysis was conducted using GraphPad Prism 8.0 (GraphPad Software, United States).

## Results

### Morphology of stap1

**Figure 1:**
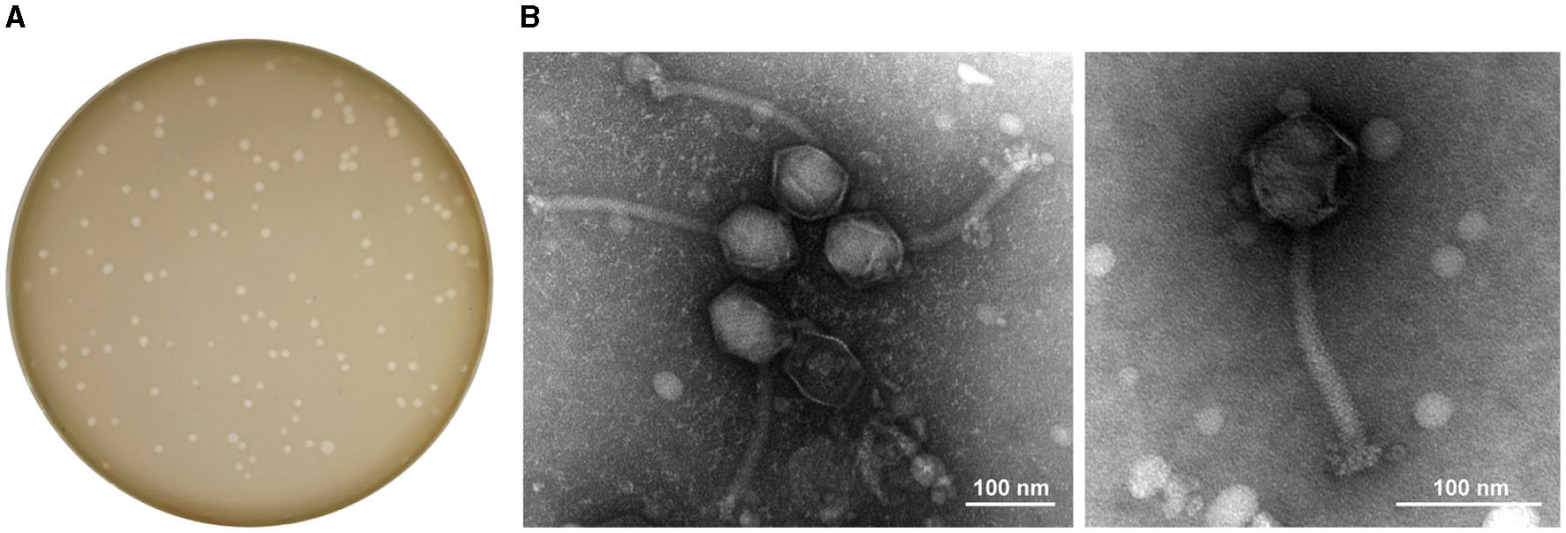
Plaques and TEM images of phage StAP1. **(A)** Morphology of phage plaques at 8 h after infection. **(B)** TEM image of phage particles. The scale bar indicates 100 nm.

Phage StAP1 was isolated from sewage collected at Southwest Hospital, and a clinical MRSA strain was used as the host bacteria. After incubation with host bacteria for 8 h, typically clear plaques with a diameter of approximately 1–3 mm were formed on the double-layer agar plate, which suggests it may be a lytic phage (Figure 1A). As shown in Figure 1B, TEM revealed that StAP1 was assigned to a member of the *Herelleviridae* family according to the International Committee on Taxonomy of Viruses (Lefkowitz et al., 2018). This phage exhibited a hexagonal head structure with an average width of ~62 nm and a tail with a length of 205 nm, which is close to other reported *Herelleviridae S. aureus* phages (Peng et al., 2019).

### Genome features and comparative genome analysis of stap1

Based on WGS, *in silico* analysis of the general genomic characteristics demonstrated that StAP1 possessed a 144,705-bp linear double-stranded DNA with 29.7% guanine-cytosine (G + C) content. According to the RAST annotation, there were 215 putative ORFs and 1 RNA gene located on the genome, of which 45 were predicted to be functional genes. Therein, virulence and antibiotic-resistant genes did not exist, implying that StAP1 is a safe candidate for phage therapy. Furthermore, there is no integrase or cleavage enzyme in the genome, which suggests StAP1 is a lytic phage.

**Figure 2:**
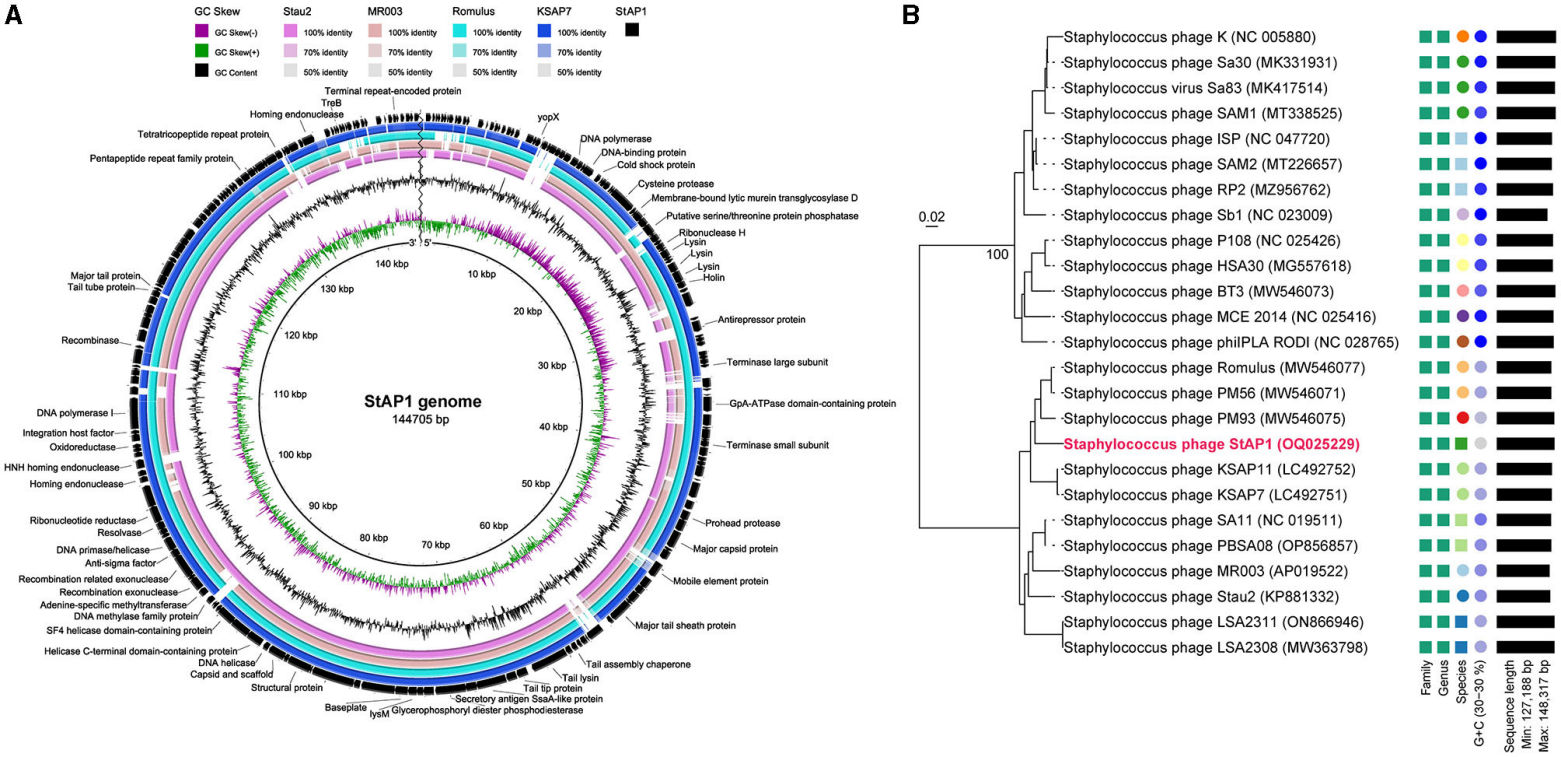
Genome features and comparative genome analysis of phage StAP1. **(A)** Comparative genomics of StAP1 with closely related phages deposited in GenBank. Concentric rings represent the similarity between the reference sequence in the inner ring and other sequences in the outer rings. Color levels indicate the result of BLASTn with a matched degree in the shared regions. The outer blank ring shows predicted ORFs. Arrows indicate the direction of transcription, and proposed functional modules have been labeled based on BLAST and domain search results. **(B)** A phylogenetic tree based on the whole genome of the phage StAP1 was generated using VICTOR. A total of 25 sequenced *Staphylococcus* phage genomes were aligned with StAP1. The accession numbers of NCBI were attached to the figure.

Multiple alignments of complete DNA sequences were conducted by comparing StAP1 with similar *S. aureus* phages deposited in GenBank, including Stau2 (KP881332), MR003 (AP019522), Romulus (MW546077), and KSAP7 (LC492751) (Vandersteegen et al., 2013; Hsieh et al., 2016; Peng et al., 2019; Kitamura et al., 2020). The query coverage between StAP1 and these phages ranged from 83 to 91%, and they shared a high similarity in genome structures (Figure 2A). To determine the relationship between StAP1 and previously reported *S. aureus* phages, a phylogenetic tree based on the WGS between StAP1 and 24 reported *S. aureus* phages was generated using the VICTOR tool. The resulting tree demonstrated that StAP1 is situated in an independent clade (Figure 2B), which suggests a relatively distant evolutionary relationship between StAP1 and other *S. aureus* phages.

### Biological profiles of stap1

**Figure 3:**
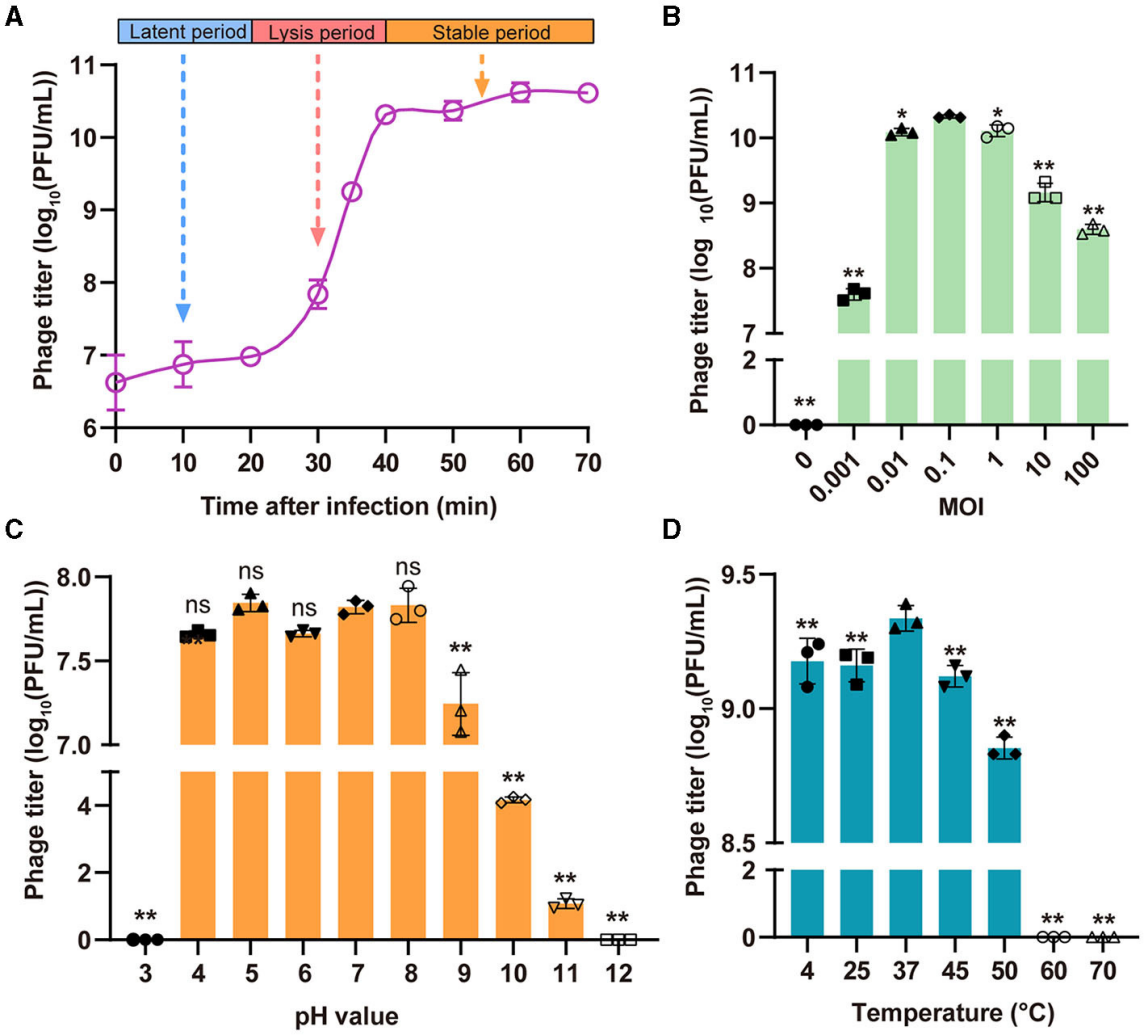
Biological characteristics of the phage StAP1. **(A)** The one-step growth curve of StAP1 infecting the host MRSA was displayed. The average values from three independent experiments are shown, with error bars indicating standard deviations. **(B)** Determination of the optimal MOI of phage StAP1; the X-axis represents different MOI values, while the Y-axis represents the corresponding phage titer. **(C)** pH stability of phage StAP1. The X-axis represents different pH values, and the Y-axis represents the phage titers incubated at 37°C for 2 h at different pH values. **(D)** Thermostability of phage StAP1. The X-axis represents different temperatures, and the Y-axis represents the phage titers incubated at different temperatures for 1 h. For **(B–D)**, data are displayed as the means plus standard deviations (SD) (error bars) from three independent experiments. *P < 0.05, **P < 0.01. ns, not significant.

The replication of phages follows three distinct phases, namely, the latent period, the lysis period, and the stable period. The one-step growth curve indicated that phage StAP1 had a latent period of 20 min, after which it experienced a burst at 20 min and peaked at 40 min. The burst size of the phage particles reached ~260 PFU per infected cell (Figure 3A). To measure the optimal MOI, the same number of bacterial cells (10^8^ CFU) were infected with various titers of phage StAP1 until the bacterial liquid was completely clarified. The phage titers in each tube were calculated, and it was identified that the samples with an MOI of 0.1 yielded the highest progeny phage titers of approximately 2.13 × 10^10^ PFU/ml (Figure 3B).

As shown in Figures 3C, D, StAP1 displayed favorable biological stability under different pH values and temperature levels. The StAP1 titers showed less variation at pH levels between 4 and 8 and exhibited partial reproduction at pH 9. However, phage particles could hardly survive at pH levels >11 or < 3. Besides, the optimal temperature for StAP1 was 37°C, and low temperatures had little effect on phage titers. However, after incubation at temperatures higher than 60°C for 60 min, most phage particles were inactivated.

### Host range determination

**Figure 4:**
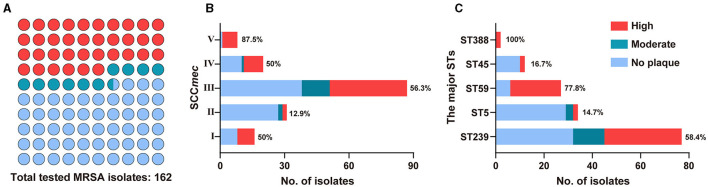
The host range of StAP1. **(A)** Plot summary showing the lytic ability of StAP1 against the total tested MRSA isolates. Fractions of the lytic rates for tested MRSA according to SCC*mec* **(B)** and the major STs **(C)** isolates. Red, ultramarine, and wathet blue modules indicate the lytic rates of high, moderate, and weak, respectively. Detailed information on the host range of StAP1 can be found in Supplementary Table 1.

To determine the host range of phage StAP1, a total of 162 clinical MRSA isolates collected from 9 teaching hospitals in six cities were subjected to sensitivity analysis. StAP1 infected 75 of the 162 MRSA strains and showed a comparatively broad-spectrum host range (Figure 4A). Furthermore, 162 strains were divided into I–IV according to SCC*mec*. It was found that ~50% of the strains belonged to SCC*mec* type III, with a sensitivity rate of 56.3% to StAP1. StAP1 showed lysis ability against all types of tested SCC*mec*, while only SCC*mec* type II was less sensitive, with a lysis rate lower than 50%. Additionally, all the tested MRSA were classified based on STs (Figure 4B). It was demonstrated that StAP1 infected the major five STs of MRSA, especially the wide-spreading ST59 and ST239 clones, with lysis rates of 77.8 and 58.4%, respectively (Figure 4C). Specifically, the community-associated (CA) MRSA ST59 clone is predominant in the dissemination across the Asia-Pacific area, while the healthcare-associated (HA) MRSA ST239 is a global pandemic clone (Gray et al., 2011; Bal et al., 2016). These results indicated that StAP1 could serve as an effective tool to contain the prevalence of MRSA clones.

### Phage therapy for *in vivo* MRSA infection

**Figure 5:**
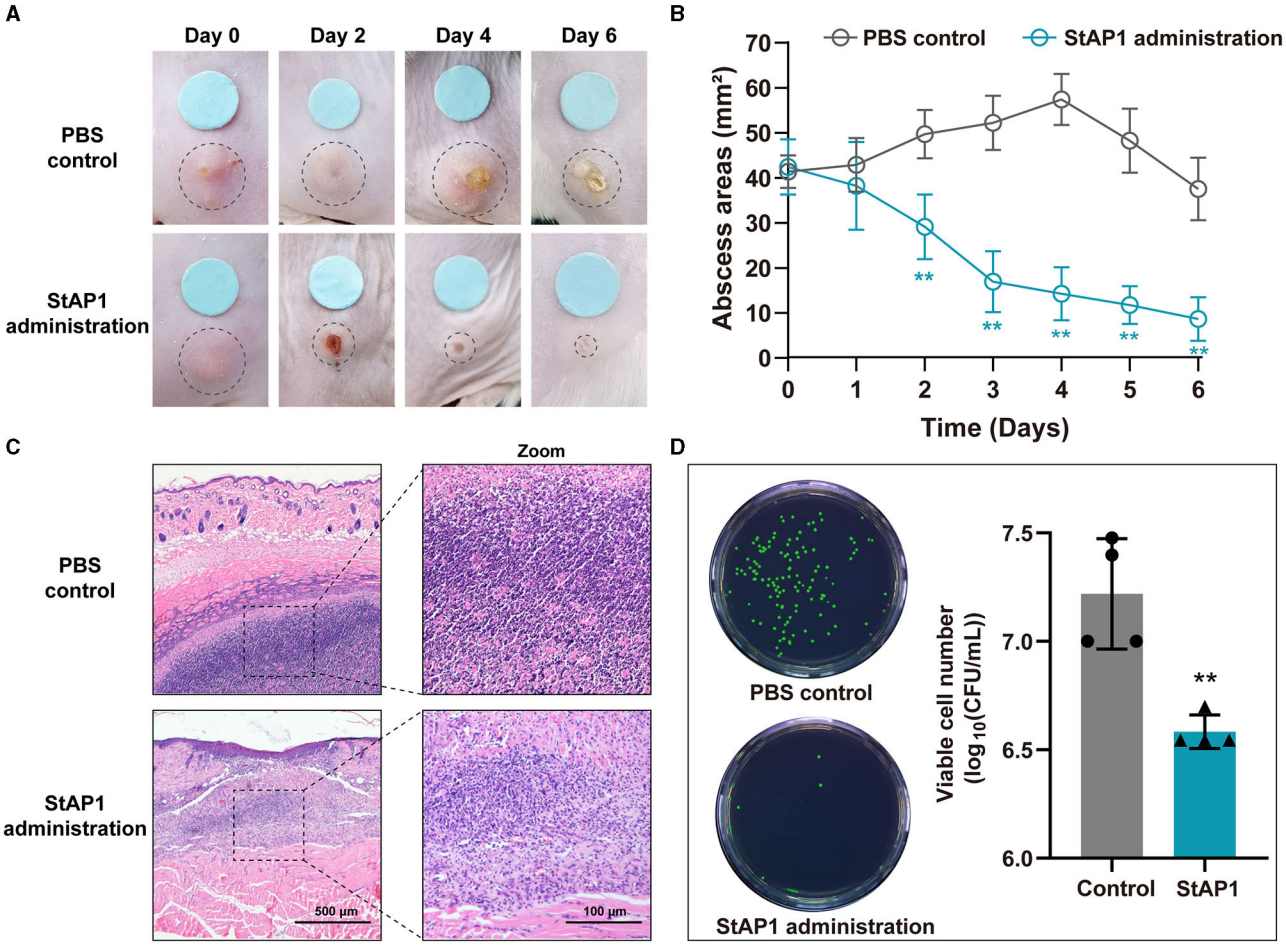
The *in vivo* antibacterial effect of phage StAP1. **(A)** Representative photographs of subcutaneous abscesses in mice treated with StAP1. **(B)** Curve of subcutaneous abscess area in mice infected with MRSA (*n* = 6). **(C)** Representative HandE staining images of infected skin after treatment. The violet circles indicate the subcutaneous abscess. The scale bar indicates 500 and 100 μm. **(D)** Quantification of infected bacteria within the subcutaneous abscess after various treatments. **P < 0.01.

Mice with formed subcutaneous abscesses were established as models of tissue MRSA infection. Compared to the control group, we observed that administration of phage StAP1 significantly facilitated the resolution of abscesses (Figure 5A). On day 6, abscess areas of < 10 mm^2^ remained with the StAP1 treatment, which was close to that of the initial day in the PBS group (Figure 5B). Thus, it can be concluded that StAP1 helps combat MRSA infection. Furthermore, histology analysis revealed that there was obvious abscess formation and inflammatory cell infiltration in the PBS group, while the degree of tissue damage was significantly reduced on account of StAP1 treatment (Figure 5C). Administration of StAP1 significantly reduced bacterial colonization (Figure 5D). These results confirmed the therapeutic effect of phage StAP1, suggesting its potential application in infectious diseases related to MRSA.

## Discussion

During this study, we successfully isolated and characterized a phage that specifically targets methicillin-resistant *S. aureus* (MRSA). The biological properties of the phage were thoroughly examined under various conditions, yielding fundamental data that can be used for future research and application of this phage. Our observations revealed the phage's robust ability to infect and destroy the *S. aureus* host, as evidenced by the formation of distinct plaques. We also utilized electron microscopy to study the phage's morphology, which exhibited typical characteristics of the *Herelleviridae* family.

In our study, we also delved into the phage's biological properties and determined the optimal MOI. We first investigated the phage's one-step growth curve, which enhances our understanding of its growth and reproduction patterns and serves as a reference for future research on this phage. Furthermore, our investigation demonstrated the excellent stability of the phage under diverse temperature and pH conditions, which is crucial for its practical application. In the treatment of infections, for instance, different sites may exhibit varying temperature and pH levels, and the phage's stability ensures its effectiveness to a certain extent. Additionally, we evaluated the host range of StAP1 and found that it possesses a broader lytic spectrum, expanding the potential applications of this phage.

Moreover, mouse experiments were conducted to validate the phage's efficacy in treating bacterial infections. These experiments revealed a significant reduction in the count of pathogenic bacteria in mice infected with *S. aureus*. This provides preliminary evidence supporting the clinical application of the phage for treating *S. aureus* infections. Nevertheless, it is crucial to acknowledge that these experiments were limited to mouse models and cannot entirely reflect the phage's effects on humans. Thus, further research and verification are necessary to ascertain its potential in human applications.

It is important to note that despite the many advantages and potential applications of phage therapy, there are still limitations and challenges in its clinical use. Factors such as the immune system and cellular barriers may influence the metabolism and diffusion rate of phages within the body (Strathdee et al., 2023). Therefore, additional research is needed to address these issues, and rigorous safety assessment and monitoring are imperative to prevent any mutations, such as gene recombination or resistance, that may alter the properties and activity of phages.

## Conclusion

This study conducted a comprehensive investigation of a phage targeting MRSA, providing fundamental data and a reference for its application and further research. Despite the limitations and challenges, phage therapy holds broad application prospects and research value. Future studies should focus on exploring the role and impact of phages *in vivo*, including research using different animal models and investigating clinical applications to verify feasibility and safety. Furthermore, delving into the genome, proteome, and biochemical metabolic pathways of phages is necessary to enhance understanding of their biological characteristics and mechanisms. The ultimate goal is to support the development of safer, more effective, and personalized treatment options, especially for drug-resistant bacteria (Pires et al., 2020; Venturini et al., 2022). We firmly believe that with the continued development and advancement of science and technology, phage therapy will increasingly play a crucial role in clinical applications, serving as an effective approach for treating drug-resistant bacterial infections.

## Data availability statement

The datasets presented in this study can be found in online repositories. The names of the repository/repositories and accession number(s) can be found in the article/Supplementary material.

## Ethics statement

The animal study was approved by the Laboratory Animal Welfare and Ethics Committee of Army Medical University. The study was conducted in accordance with the local legislation and institutional requirements.

## Author contributions

YuL: Data curation, Software, Writing–original draft. YiL: Conceptualization, Data curation, Software, Visualization, Writing–original draft. BL: Writing–review and editing, Formal Analysis, Software. JL: Writing–original draft. LW: Writing–review and editing. LZ: Writing–review and editing, Project administration, Supervision. YLi: Writing–review and editing, Validation. QZ: Conceptualization, Funding acquisition, Methodology, Resources, Supervision, Writing–review and editing.
